# Evaluation of Vasculogenic Factors in the Developing Embryo at Weeks Five and Seven With a Special Focus on CD133 and TIE2 Markers

**DOI:** 10.7759/cureus.60353

**Published:** 2024-05-15

**Authors:** Larisa Cristina Tomescu, Ioan Sas, Simona Sarb, Anca Maria Cimpean

**Affiliations:** 1 Obstetrics and Gynecology, Victor Babeş University of Medicine and Pharmacy, Timișoara, ROU; 2 Microscopic Morphology/Histology, Victor Babeş University of Medicine and Pharmacy, Timișoara, ROU

**Keywords:** human embryo, endothelial progenitor cells, vasculogenesis, tie2, cd133

## Abstract

Background

Human embryo vasculogenesis (blood vessel development starting from endothelial precursors) includes the ability of mesenchymal cells and pluripotent stem cells to differentiate into endothelial cells. Quantification of endothelial progenitor cells is difficult to assess during the early steps of human embryo development due to several factors, especially due to the paucity of human embryo tissue which is usually discarded after early-stage pregnancy abortive methods. CD133 (Promimin-1) is a general marker of progenitor cells, but combined with other endothelial markers such as CD34, it may identify endothelial progenitor cells during embryonic development. CD34 immunohistochemistry was previously performed by our team to identify human embryo capillaries and comparatively assess microvessel density between different human embryonic tissues. TIE2 is an angiopoietin receptor strongly involved in the newly formed blood vessel maturation due to its expression in some mesenchymal precursors for future pericytes. CD34 assesses the presence of endothelial cells but its single use does not evaluate the endothelial progenitor state as CD133 may do nor vessel maturation as TIE2 may do. Data about the dynamics of CD133/TIE2 expression in the early stages of human embryo development are scarce. Hence, in this study, we aimed to comparatively assess the dynamic of CD133+ endothelial precursors and TIE2 expression on five and seven-week-old human embryonic tissues with a special emphasis on their expression on embryonic vascular beds.

Methodology

CD133 and TIE2 immunohistochemistry was performed on five and seven-week-old human embryonic tissues followed by their quantification using the Qu Path digital image analysis (DIA) automated method.

Results

CD133 and TIE2 showed divergent patterns of expression during the initial phases of human embryonic development, specifically in the vascular endothelium of tiny capillaries. The expression of CD133 in endothelial cells lining the perfused lumen gradually decreased from five to seven-week-old embryos. It remained expressed with greater intensity in cells located at the tip of the vascular bud that emerged into pre-existing capillaries. TIE2 was much more specific than CD133, being restricted to the level of the vascular endothelium; therefore, it was easier to quantify using digital image analysis. The endothelium of the embryonic aorta was an exception to the divergent expression, as CD133 and TIE2 were consistently co-expressed in the seven-week-old embryo. The Qu Path DIA assessment increased the accuracy of CD133 and TIE2 evaluation, being the first time they were quantified by using automated software and not manually.

Conclusions

High heterogeneity of CD133 and TIE2 was observed between five and seven-week-old embryonic tissues as well as between different embryonic regions from the same gestational age. The unique finding of CD133/TIE2 co-expression persistence inside aortic endothelium needs further studies to elucidate the role of this co-expression.

## Introduction

Embryonic tissue and embryonic appendages have always been controversial microscopic aspects in terms of the existence and functionality of certain histological structures during fetal development [1] and the maternal-fetal interrelationship [2]. The morphological aspects of placental structures are sometimes overlooked even in high-risk or prematurely terminated pregnancies [3], which negatively impacts the subsequent elucidation of causes leading to these undesirable events. Embryonic organogenesis includes the development of vascular networks adapted to each organ individually [4]. The collection of human embryos is extremely difficult. They are usually obtained from elective pregnancy terminations or those caused by various pathological conditions [5]. Immunohistochemical and molecular microscopic analyses of embryonic development precursor stages involve extracting the entire embryo, a maneuver extremely difficult to perform during abortion.

The vascular network represents a key point in the embryonic development of all embryonic organs, representing a critical moment with heterogeneous cellular components that will later determine the normal or abnormal development of embryonic organs [6]. Vascular networks in different organs develop differently. The mechanisms underlying the development of vascular networks are incompletely described in human embryonic tissues due to strict ethical complications associated with using human embryonic tissues [7]. Mathematical models for developing embryonic vascular networks are heterogeneous from one organ to another and underlie the application of morphometric studies forming vascular networks. In addition to the limited microscopic aspects described in the literature regarding embryonic vascular characteristics, few studies have been conducted on embryonic appendages, namely, the umbilical cord and placenta [8].

Based on the above, this study attempted to elucidate lesser-known aspects of microscopy and molecular analysis of embryo-fetal structures during their early and late developmental stages. This study addressed fetal embryonic structures both from a clinical and a microscopic and molecular perspective, i.e., conventional microscopic analysis and digital image analysis (DIA) techniques of developing embryonic vascular networks in weeks five to seven in different primordia of embryonic organs.

Embryonic development of blood vessels involves cellular and molecular mechanisms, including the ability of mesenchymal cells and pluripotent stem cells to differentiate into endothelial cells participating in vascular endothelium formation. In addition, other factors involved in angiogenesis such as TIE2 (the receptor for angiopoietin) represent significant steps in assessing embryonic vascularization [9].

CD133 is a general marker for progenitor cells including endothelial and hematopoietic cells. CD133 is highly expressed in the endothelial progenitor cells during embryonic development, but its expression timing during embryonic and fetal staging is mostly unknown for human embryonic tissues [10]. TIE2 is a receptor for angiopoietin which starts to be expressed during vessel maturation, as well as inside the hematopoietic islands during the early development of the human embryo. CD133 and TIE2 expression is not fully characterized in human embryonic tissue. Both CD133-positive cells and TIE2-positive cells are not only crucial in embryonic vascularization but also in postnatal life, being factors involved in tumor vascularization or tumoral angiogenesis [10].

Quantification of CD133-positive cells on human embryonic tissues has been discussed concerning CD34 expression, suggesting that this antibody panel would characterize hematopoietic stem-like cells during embryonic development. Hence, studying CD34+/CD133+ cells enabled mapping hemogenic stem cells [11] within fetal endothelium which later assisted in quantifying hematopoietic stem cell maturation degree as well as their functionality.

Paradoxically, CD133+ cells have been scarcely studied concerning their participation in vasculogenesis and embryonal angiogenesis. In the literature, 11 articles have mentioned CD133+ cell involvement in human embryo vasculogenesis while 57 referred to their role in human embryo angiogenesis. Based on existing data, CD133+ cells are responsible for emerging endothelial precursors for both lymphatic endothelial cells as well as blood vessel endothelium [12].

It appears that CD133+ is most commonly used in identifying endothelial progenitors [13] during embryonic development together with CD34 and other endothelial markers. Moreover, CD133+ cells are extensively studied as cells participating in vascular repair at cardiac, hepatic, and renal levels during embryonic, fetal, and postnatal angiogenesis and vasculogenesis. Scarce data are available concerning TIE2 expression in the early stages of human embryo development. However, there is no quantification of the dynamic expression and mutual interrelation of CD133 and TIE2 in the early stages of human embryo development.

Based on the above evidence, we aim to investigate the interrelation of CD133 and TIE2 in human embryo vasculogenesis and the mesenchyme surrounding different embryonic organs primordia along with their expression inside these primordia. We mainly focused on CD133 and TIE2 expression at the embryonic vascular network as well as in other embryonic tissues such as nervous tissue from the spinal cord and brain, mesenchymal tissue around each target organ, heart, aorta, thymus, trachea, peripheral nerves, and lung in five and seven-week-old human embryos using immunohistochemistry and automated quantification based on Qu Path DIA.

## Materials and methods

This study was conducted in a collaborative effort between the Histology Department within the Faculty of Medicine and Pharmacy Victor Babeș Timișoara and the Municipal Emergency Hospital Timișoara. This collaboration allowed for a diverse range of expertise and resources for the research. Before the study commenced, it was thoroughly reviewed and approved by the Ethics Committee of the Victor Babeş University of Medicine and Pharmacy in Timișoara (approval number: 69/2020) and the agreement of the Timisoara Municipal Emergency Clinic Hospital to access the hospital’s database (approval number: 81/12.12.2022) in compliance with the provisions of regulation number 679/2016 and the legislation in force regarding the protection of personal data and the law 46/2003 regarding patient prescriptions. Written informed consent was obtained from patients before the surgical procedure. In the informed consent, it was stated that the patients agreed that their tissues may be used for research purposes. The focus of the study was specifically on the early stages of embryonic development, specifically weeks five to seven. This period is critical as significant development and differentiation occur in the embryo during this period. The researchers chose this timeframe to gain insights into the fundamental processes that drive embryonic vessel development. This examination was conducted from multiple perspectives, including clinical, microscopic, and molecular viewpoints.

Human embryo sample collection and management

Following a legal abortion, 10 whole human embryos aged five weeks and seven embryos aged seven weeks were collected following the ethical standards. Carnegie stages were applied to determine the gestational age. Inclusion criteria were (1) clinically healthy pregnant women; (2) living human embryos with a gestational age of five and seven weeks; (3) extraction of the whole human embryo with intact gestational sac; and (4) informed consent agreeing to use embryonic tissues for research purposes. Exclusion criteria were (1) pregnancies with associated pathologies; (2) incomplete gestational sac or human embryo; (3) lack of informed consent of the patients for the use of their tissues for research purposes; and (4) human embryos with suspicion of not meeting the selected gestational age of the present study.

Primary processing of the human embryonic tissues

Following 48 hours of fixation in 10% buffered formalin and paraffin embedding, the whole embryo was sectioned into 3 µm serial sections. After staining each 10th slide with hematoxylin and eosin for morphological assessment, the slides were sequentially selected for the next steps of the studies. Selection criteria included (1) proper staining with hematoxylin and eosin; (2) sagittal section of the whole embryo; and (3) no artifacts as folds or disrupted tissue sections. All tissue sections meeting these criteria were immunostained with CD133 and TIE2 antibodies.

Immunohistochemistry

Immunohistochemistry was performed using Leica Bond-Max (Leica Biosystems, Newcastle Upon Tyne, UK) Autostainer on 3 μm thick sections. Unmasking was performed using Novocastra Bond Epitope Retrieval Solution 1 and 2, pH6 and 9 solutions (Leica Biosystems, Newcastle Ltd., Newcastle Upon Tyne, UK). Inhibition of endogenous peroxidase was performed using 3% hydrogen peroxide for five minutes. The immunostaining procedure was applied using CD133 rabbit anti-human polyclonal antibodies (Santa Cruz Biotechnology UK, 30-minute incubation time at room temperature) for vessel endothelium progenitors and TIE2 rabbit anti-human polyclonal antibodies (Santa Cruz Biotechnology, UK, 30-minute incubation time at room temperature) for blood vessel maturation. Visualization systems such as Bond Polymer Refine Detection System DAB were used for completing the immunohistochemical procedure. CV Mount (mounting medium from Leica Biosystems, Newcastle Ltd., UK) was used as a permanent mounting medium for immunohistochemistry-stained slides.

Hematoxylin and eosin-stained slides and immunohistochemistry evaluation

The hematoxylin and eosin-stained slides and immunohistochemistry samples were scanned and analyzed using a Grundium OCUS 20 Microscope (Grundium, Tampere, Finland) and saved in the Case Centre Slide Library as svs files (3DHistech, Budapest, Hungary). The quantification of CD133 and TIE2 was performed on the entire histological section but also on different types of embryonic tissues. A project was initiated by importing all slides into QuPath version 0.4.3, an open-source platform for analyzing microscopic slides in bioimage analysis. The slides were then examined using integrated software and its add-ons, including Fiji and Vascular Analysis, to accurately evaluate CD133 and TIE2 expression in human embryo vessels and mesenchymal tissue. Briefly, we selected three to five regions of interest. The DIA procedure commenced by performing a pre-processing step which involved the estimation of stain vectors. Next, the analysis proceeded by selecting a positive cell detection option and configuring cell parameters and intensity parameters for cell detection. The detected image was configured to calculate the sum of optical densities using a pixel size of 0.5 μm and a cell expansion of 1.988 μm, excluding the nucleus. The intensity threshold parameters consisted of a score compartment and three threshold levels, i.e., weak (+1, highlighted in yellow), moderate (+2, highlighted in orange), and strong (+3, highlighted in red). Cells picked in blue were regarded as negative. During automated scoring, the QuPath software provided the following information: the number of cells, the percentage of positive and negative cells, the density score, the intensity score, and a combined stromal score (SS), which is similar to the Allred score. The SS combines the intensity and density of positive detected cells. In addition, the final evaluation completed using QuPath analysis incorporated an H-score.

## Results

The quantification of the two markers, CD133 and TIE2, was performed separately across all stages of blood vessel development, starting from cells isolated from embryonic mesenchyme and continuing with stages of vascular cords, vascular tubes, and perfused vessels (Table 1).

**Table 1:** Expression of AC133 and TIE2 in five and seven-week-old embryos. + = low intensity; ++ = medium intensity; +++ = high intensity; - = absence

Organs	Isolated cells			Vascular cords			Vascular tubes			Perfused vessels		
	CD133+	TIE2 +	CD133+/TIE2-	CD133+	TIE2 +	CD133+/TIE2-	CD133+	TIE2 +	CD133+/TIE2-	CD133+	TIE2 +	CD133+/TIE2-
Nervous tissue from the spinal cord and brain	+++	-	++	++	++	++	-	++	-	-	+++	+
Mesenchymal tissue	+++	+	++	++	+++	+	-	+++	+	-	+++	-/+
Heart	-	+++	-/+	-	-/+	-/+	-	+++	-/+	-	+++	-/+
Aorta	+++	+++	+++	++	+++	++	+++	++	++	+	+++	+
Thymus	+	-	+	+	-	+	-	-	-	-	-	-
Trachea	+++	+	+	+	++	+	-	+++	-	-	+++	-
Peripheral nerves	++	-	+	-	++	-	-	++	-	-	+++	-
Lung	+++	++	+	+	+++	-/+	-	++	-	-	+++	-/+

Expression of AC133 in five and seven-week-old embryos

**Figure 1:**
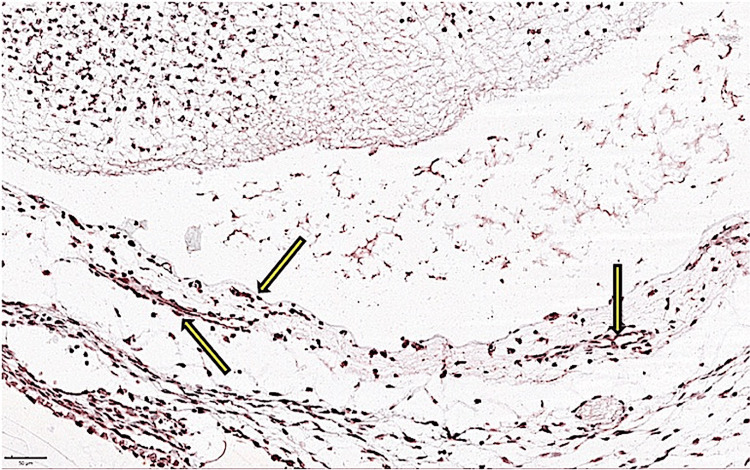
The presence of vessels delimited by CD133-positive endothelium in the mesenchymal tissue around the brain tissue primordium. CD133-positive vessels were observed in different stages of development from cord-like agglomerations to patent, lumen-perfused vessels.

In the embryo at five weeks, the expression of AC133 was present both in the organ primordia and at the level of vessels in the mesenchymal tissue that surrounded the organ primordia. In these five-week-old embryos, the intensity of expression had significantly increased in vessels within the mesenchymal tissue, while this expression was weakly and inconsistently detected within the tissues of organ primordia, especially in nervous tissue in structures with vascular morphology. A high density of AC133+ cells was organized in the form of cords at the level of primitive nervous tissue in the cerebral hemispheres where AC133+ cells were detected at the tip of the vascular bud (Figure 1). During this period, in vessels within the nervous tissue that showed perfusion (erythrocytes), the expression of AC133 was negative in endothelial cells that delimited the perfused lumen. Most vessels that were delimited by AC133+ endothelial cells were distributed in the mesenchymal tissue around the neural primordium. Furthermore, at the level of the head and neck, the expression of CD133 in vascular structures was present in the mesenchymal tissue of future differentiated mandibular tissue. It was noteworthy that as the tissue had become more differentiated, the density of CD133+ cells at the level of the vascular endothelium had decreased significantly.

In embryos at five and seven weeks, a reduced number of vascular islands were observed within the embryonic mesenchymal tissue. A peculiarity of CD133 expression was noted at the level of the aorta where the aortic endothelium was intensely positive for CD133 at seven weeks of intrauterine life. Moreover, large vessels from the adventitia of the aorta presented negative CD133 endothelium while isolated cells with a fibroblast-like appearance had persisted in this aortic layer.

A high density of CD133+ cells was also observed in the positive adventitia of the trachea where inconsistent expression of CD133 was observed at the level of the endothelium of adventitial vessels. Compared to the vessels existing in the aortic wall, we observed CD133 expression in the vessels of the lamina propria of the trachea but not in the submucosa. Most likely these vessels were of the lymphatic type. The vessels in the adventitia of the trachea were rarely and inconsistently positive for CD133 in the sense that CD133+ endothelial cells had alternated with CD133- endothelial cells. In the seven-week embryo, we did not observe vascular islands that contained CD133+ cells.

In the seven-week embryo, the endothelium of large capillary vessels was negative, while capillaries at the level of the connective trabeculae of the thymus retained a weak reaction to CD133. Inside the thymic parenchyma, we noted CD133 expression at the level of the thymic stroma and very rarely at the endothelial level. Focally, Hassall’s corpuscles were weakly positive for CD133, especially those in the early stages of development and the epithelial cells at the periphery of Hassall’s corpuscles. At the level of the cortex, subcapsular endothelial cells also presented a positive reaction to CD133.

At the level of the esophagus, CD133 was positive in isolated cells from the vessels of the esophageal wall, especially at the level of the submucosa. The vessels in the muscularis as well as those in the lamina propria were already mature vessels at seven weeks, perfused and without the expression of CD133 inside the endothelium. Taking into account the morphology of the vessels analyzed, we could affirm that CD133+ cells were found in venous-type vessels and compared to the endothelium of arterial-type vessels. This was also noted at the level of the other organs, not being specific to the esophageal wall.

Expression of AC133 in vessels from perinervous mesenchymal tissue (around the spinal cord) showed positive expression for CD133 in all stages of development, starting from cells constituting the mesenchymal vascular islands to perfused vessels. The expression was constant until the perfused vessels, with the highest intensity of expression observed in the perinervous mesenchymal tissue, suggesting the presence of an active process of vasculogenesis and angiogenesis, probably closely correlated with the activity of the nervous tissue. Mesenchymal cells in the mesenchymal tissue were gradually negative for CD133, in the sense that those cells differentiated into fibroblasts (fibrocytes) were completely negative, while stellate cells in the differentiated mesenchymal tissue presented a positive reaction to CD133.

With the perfusion of blood vessels, the intensity of CD133 expression gradually decreased in endothelial cells that lined the perfused lumen but remained expressed with increased intensity in cells at the tip of the vascular bud that emerged into pre-existing vessels.

In the seven-week embryo, the expression of CD133 had a lower intensity except for the adventitia of large vessels and tubular organs, where the intensity of CD133 at the level of the vascular endothelium was similar to that of the five-week embryo. At the level of the aorta, the aortic endothelium was constantly positive for CD133, suggesting incomplete maturation of the endothelium at seven weeks of intrauterine life, supporting the hypothesis that the aortic endothelium is a source of hematopoietic stem cells.

At the level of peripheral nerves, CD133 was expressed only peripherally in the cells of the epineurium inconsistently.

In the thymus, CD133-positive cells were present not in the vascular endothelium but as isolated cells in the thymic parenchyma. However, the perithymic connective tissue presented AC133-positive cells in small vessels, the endothelium of large vessels being negative.

At the pulmonary level, in the seven-week embryo, CD133+ cells were present at the endothelial level predominantly in interalveolar capillaries.

Expression of TIE2 in five and seven-week-old embryos

Unlike CD133, which was weakly and inconsistently positive in vascular structures within the embryonic nervous tissue in the five-week embryo, TIE2 was present with moderate intensity but outlined the vascular structures within the nervous parenchyma. TIE2 was much more specific than CD133, being restricted to the level of the vascular endothelium, and for this reason, it was easier to quantify using DIA. At the level of the nervous test, the endothelium’s expression of TIE2 was observed both in endothelial cells that lined the lumen of large perfused vessels and in small vascular structures with a cord or tube aspect. Paradoxically, the intensity of TIE2 expression in vessels in the nervous tissue was weak. This aspect was confirmed by the H-score obtained using the QuPath system. The mesenchymal tissue around the developing nervous tissue included a rich network of TIE2+ vessels with a tendency to form vascular networks (interconnected endothelial extensions). TIE2-positive structures with a cord aspect without lumen predominated, followed by small caliber tubes (most likely capillaries) not perfused. With the perfusion of TIE2+ vessels, the intensity of TIE2 expression decreased progressively, and a significantly increased number of TIE2+ cells with a tendency to organize in cords and form networks was observed in the connective tissue in the developing nervous tissue.

In the connective tissue, during the process of differentiation at the level of the cells of the mandibular zones, TIE2+ structures were also observed, similarly organized with those at the level of the perinervous connective tissue, but in this region, TIE2-positive structures with a cord aspect predominated.

Very few of these had delimited a well-defined lumen. A particular aspect was represented by the expression of TIE2 at the level of the connective sheaths of the nervous tissue. Capillary vessels from the structure of the choroid plexuses were negative for TIE2, while vessels from the internal area of the dura mater presented a weak/moderate reaction to TIE2.

If for the nervous test, the expression of TIE2 had been intense and constant, the expression of TIE2 at the level of other organs was extremely heterogeneous.

At the pulmonary level, in the seven-week embryo, the expression of TIE2 was not noted in the vascular structures from the peribronchial interalveolar parenchyma. On the other hand, the endothelium of the aorta was positive for TIE2 with an intensity similar to that of CD133. There were no observed endothelial expressions at the level of the periadventitial aortic vessels and no isolated TIE2+ cells. Moreover, TIE2 was negative in peripheral nerves both at the endothelial level and at the level of their parenchyma.

The thymus was also negative on immunostaining with TIE2.

At the level of the esophagus, vessels from the lamina propria and submucosa were negative for TIE2, while vessels from the esophageal adventitia and those distributed at the limit between the internal and longitudinal external layer retained a positive reaction to TIE2.

Comparative results of digital image analysis applied for the quantification of TIE2 and CD133 in human embryos of five and seven weeks were performed. CD133/TIE2 comparative analysis of the parameters was quantified by the QuPath DIA system.

**Figure 2:**
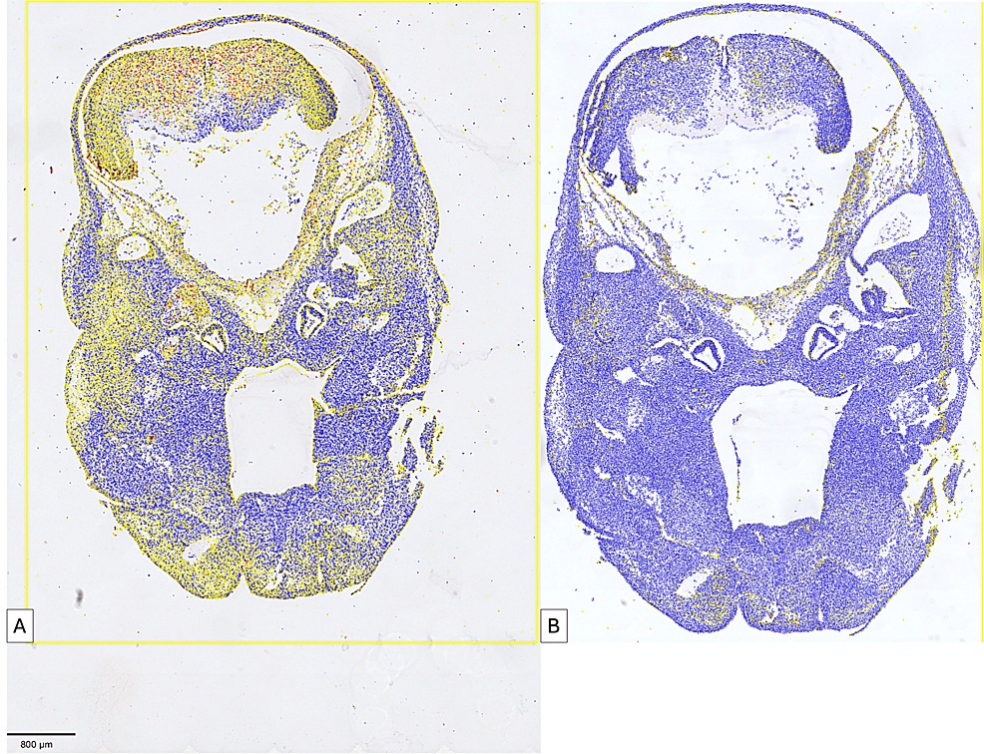
Comparative analysis of CD133 (A) and TIE2 (B) expression in a five-week-old embryo. Note the divergent expression of the two markers. At five weeks, CD133 predominates with low-to-moderate intensity both in the mesenchymal tissue and the tissues differentiated from the nervous tissue in the cephalic region.

As seen in Figure 2, the global evaluation of CD133 (Figure 2A) and TIE2 (Figure 2B) revealed a divergent expression of the two markers. The signals detected by the QuPath system were evaluated differently according to intensity by the presence of four color signals: (1) blue signal - negative cells; (2) yellow signal - cells with weak signal intensity; (3) orange signal - cells with medium immunohistochemical signal intensity; and (4) red signal - cells with high intensity of immunohistochemical expression.

**Figure 3:**
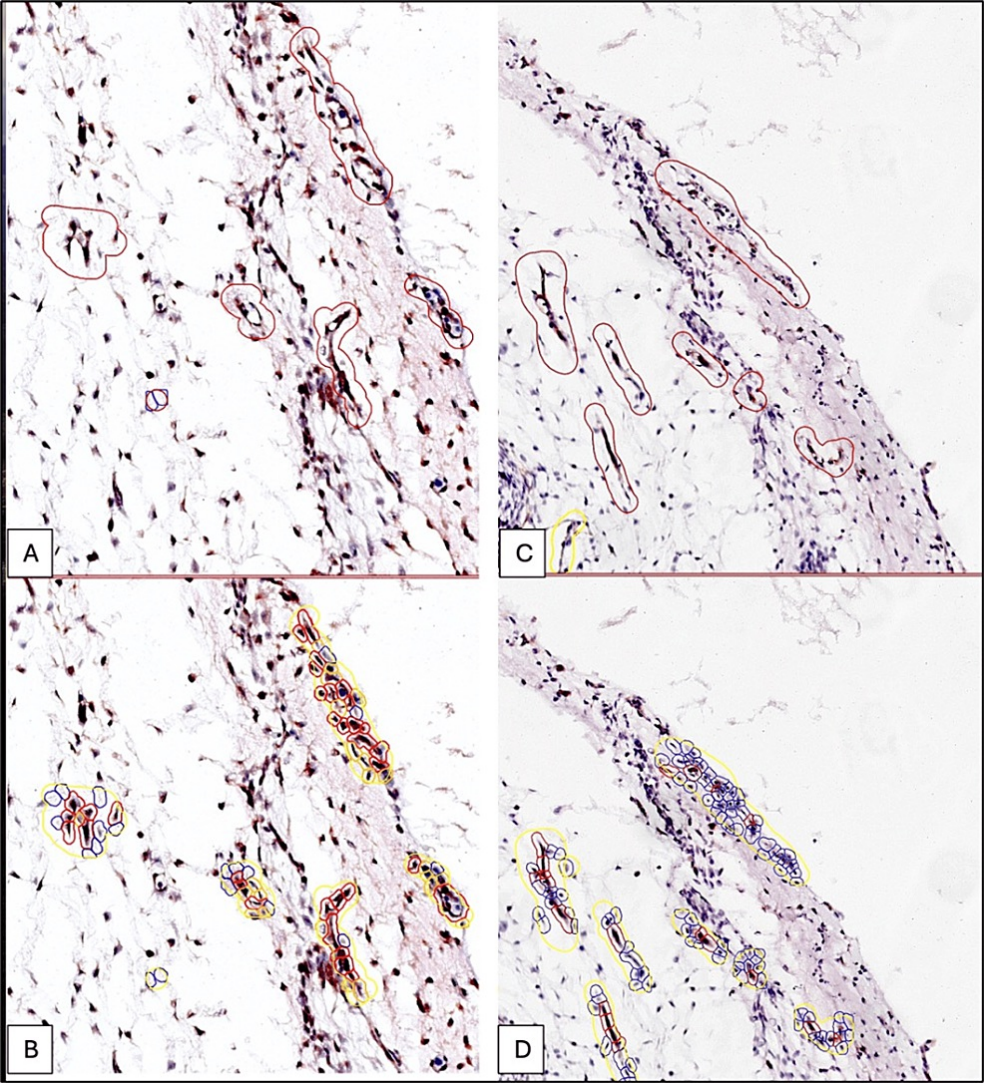
Comparative expression of CD133 (A, B) and TIE2 (C, D) in a five-week-old embryo mesenchyme. Note that CD133 expression (A, B) has a considerably higher number of red (intense positive) signals compared to TIE2 (C, D) at the same gestational age.

As can be seen from Figure 3, the density of CD133+ cells was significantly higher compared to the density of TIE2+ cells in the five-week embryo. Regarding the expression of CD133, we identified a moderate density of red signals at the level of the primordium of the nervous tissue of the future brain. CD133 expression was not restricted to the vascular level but was also detected in progenitor cells from the primitive nervous tissue, which denoted the ability of AC133-positive cells to differentiate into multiple cell lines.

**Figure 4:**
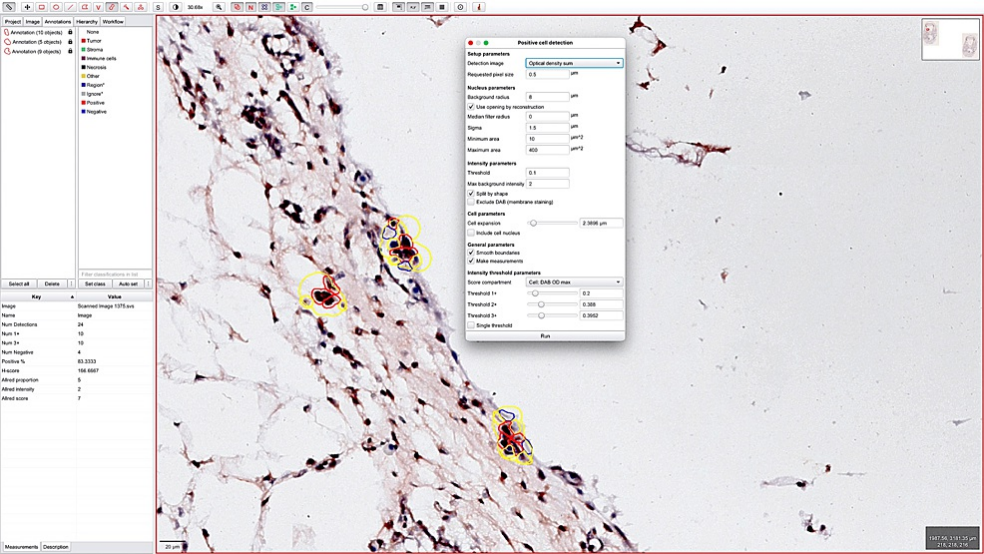
Digital image analysis detected the presence of precursor blood elements within the vessels delimited by AC133-positive cells.

At the vascular level, the maximum density of CD133-positive cells was quantified by DIA in undifferentiated or weakly differentiated mesenchymal tissue toward hematopoietic precursors predominantly into the nervous tissue surrounding mesenchyme (Figure 4).

**Figure 5:**
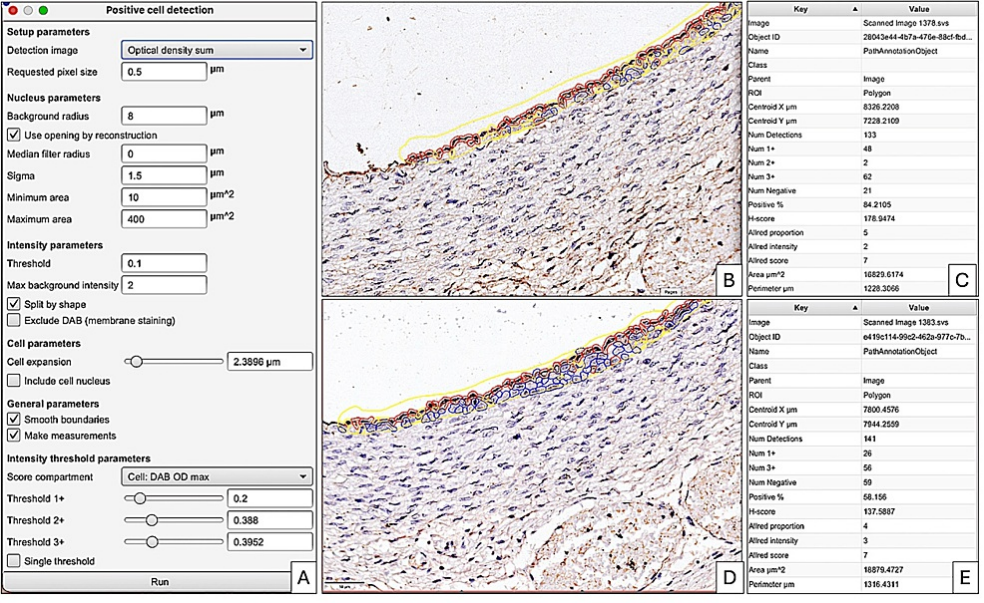
Parameter criteria set for the evaluation of CD133 (B) and TIE2 (D) expression in the aortic endothelium at seven weeks of gestation. Note that for both markers it predominated the red contour around positive cells (B, D). This means that both markers have the same intensity and density expression in the endothelium of the aorta based on the Allred score combining the intensity and density of positive cells (C, E).

The only structure where the endothelial expression of CD133 and TIE2 was similar in the five and seven-week embryo was the developing aortic endothelium, which maintained its TIE2 expression with the same intensity and density as the CD133 expression in the aorta of the seven-week embryo (Figure 5).

## Discussion

CD133 is well known as a marker of progenitor cells with the capacity to differentiate into various cellular lines in all organs [14]. It is intensely expressed at the level of mesenchymal cells during embryonic development. CD133 has been extensively studied, especially in identifying cells with potential malignant cells, with progenitor potential being responsible for the phenomenon of epithelial-mesenchymal transition very often encountered in various types of malignant tumors [15-17]. As intensely as it was studied in malignant lesions, the expression of CD133 in the early stages of human embryonic development is less studied in human embryonic tissue. Moreover, hematopoietic stem cells are considered progenitor cells for the differentiation of other cellular types [18,19]; however, CD133 expression at the level of embryonic endothelial cells is not well-known. In the initial stages of embryonic development, CD133 is expressed both in progenitor endothelial cells and hematopoietic stem cells, but their dynamics depending on the age of the embryo have been scarcely addressed. This study confirms the expression in the five-week embryo of CD133 both at the level of endothelial cells in the vessels of undifferentiated mesenchymal tissue and in hematopoietic islands observed in this type of mesenchymal tissue. Furthermore, our study demonstrates the increased intensity of expression in vessels from undifferentiated mesenchymal tissue and the decrease until the disappearance of the expression with tissue differentiation, so that in nervous tissue, CD133 presented nuclear expression in neuronal progenitor cells but was not detected in endothelial cells of intracerebral vessels. In the seven-week embryo, CD133 expression significantly decreased, and the only area where it remained intensely positive was the endothelium of the aorta. The role of CD133+ cells in embryonic development was realized in close correlation with their capacity to differentiate toward endothelial cells also reporting a significant involvement in the regeneration of the capillary glomerulus at the level of the renal corpuscle [20]. Most studies have been conducted on CD133+ cells isolated from the umbilical cord [21-23], which were isolated and cultivated with the possibility of differentiating into endothelial cells. Data on the heterogeneity of CD133 expression at the level of the vascular endothelium of the embryo in the early stage of development are limited, leading to a partial understanding of its role in the early stages of the development of embryonic vessels. For this reason, we consider it useful to study it comparatively in five and seven-week-old embryos. In contrast to CD133, TIE2, a well-known factor involved in regulating angiogenesis and vascular maturation [24], seems to have a divergent expression from that of CD133. Weakly expressed in the five-week embryo, TIE2 was intensely expressed in the endothelium of the vessels of the seven-week embryo, which suggested the stabilization of the embryonic vascular network. The study of TIE1 and TIE2 is extensively conducted at the level of the placenta [25,26] but to a lesser extent on embryonic tissues.

In a study by Sârb et al., the morphological expression of TIE2 in five and seven-week embryos was demonstrated. They showed that the expression of TIE2 was intense in vessels with immature morphological characteristics from the perimedullary and perinervous mesenchyme, an aspect that was maintained in both five and seven-week embryos [9]. By performing a differentiated analysis between CD133 and TIE2, it was noted that the only place where the expression of the two markers was convergent and sustained was at the level of the aortic endothelium. In the seven-week embryo, the entire aortic endothelium was positive for both markers with the same intensity. This expression of the two could have been critically discussed from several points of view. The data on the combined expression of CD133 and TIE2 at the level of the aortic endothelium supported the preservation of the hemogenic function of the aortic endothelium with the ability to generate both endothelial cells and hematopoietic precursors. Previous studies described CD133 expression at the level of the hemogenic aortic endothelium in the aorta-gonad-mesonephric area at six weeks post-fertilization [10] but through other molecular techniques than those of protein-level and microscopic expression. If for CD133 the expression at the endothelial level was certified and validated previously by other studies, the expression of TIE2 was less validated at the level of the aortic endothelium. A study by Jiang et al. [27] investigated the expression of TIE2 at the level of the gonads at an age similar to the seven-week embryo and reported that this expression could not be detected in vascular endothelial cells in the gestational interval between three and 12 weeks. The data contradicted these previously published results in the sense that TIE2 had been intensely expressed at the endothelial level both in small mesenchymal vessels and in the aortic endothelium at the level of the thoracic aorta in the seven-week embryo. Other studies on TIE2 expression at the endothelial level have been conducted on an animal model [28], mainly avian, but not on human embryonic tissue. In the avian model, TIE2 was expressed at the level of the endothelium of embryonic veins and very weakly and inconsistently at the level of arterial endothelium. This contradicted the results which had demonstrated the intense and constant expression of TIE2 in the seven-week human embryo. Due to the lack of data in the literature regarding TIE2 in human embryonic tissues, it was impossible to compare the data with other data in the literature. There was only one recent article in which the expression of TIE2 was marked in CD31-positive cells, but these results were obtained in vitro, unlike the data from human tissues. The endothelial cells that expressed TIE2 in the study by Betucci et al. [29] were isolated from the level of the umbilical cord veins (HUVEC) (with venous origin) but were capable of generating cord lines and cordon-like structures. If TIE2 expression in the aortic endothelium had been mentioned in the literature in correlation with the mechanisms of development of arteriosclerosis in the murine model [29], with the mention that these cells considered progenitors had vasculogenic properties.

As with any research, this study has several strenghts and limitations. The detections of differences between CD133 and TIE2 from weeks five and seven of human embryo development highlighted an intensive vasculogenesis process followed by a rapid maturation of the embryonic vascular bed. Another strength of this study was the finding about the persistence of CD133 expression and its co-expression at the human embryo aorta in the seventh week of gestation. This finding sustains the persistence of endothelial progenitor cells inside aortic endothelium most probably a source for hemogenic endothelium. The differential expression of CD133 and TIE2 in several human embryo organs, with special emphasis on the rapid dynamic of their expression between weeks five and seven of gestation at the level of vital organs as nervous tissue.

The study, while providing valuable insights into the expression of CD133 and TIE2 in the early stages of human embryonic development, had several limitations. One of the main limitations was the lack of data in the literature regarding the co-expression of CD133 and TIE2 at the aortic endothelium of the human embryo, which made it impossible to identify the function of this co-expression. The study was also limited by the fact that most of the research on TIE2 expression at the endothelial level was conducted on an animal model, mainly avian, but not on human embryonic tissue. This discrepancy could have potentially influenced the results. Furthermore, the study was unable to compare its data with other data in the literature due to the lack of studies on TIE2 in human embryonic tissues. Finally, the study was based on the analysis of embryos at only two stages of development (five and seven weeks), which might not fully represent the dynamics of CD133 and TIE2 expression throughout the entire embryonic development.

## Conclusions

This study demonstrates the divergent expression of CD133 and TIE2 in the early stages of human embryonic development at the vascular endothelium level in small embryonic capillary-type vessels as well as in large aortic-type vessels. An exception to this divergent expression is by the endothelium of the embryonic aorta, where the persistent co-expression of CD133 and TIE2 was demonstrated in the seven-week embryo. Due to the lack of data in the literature regarding the co-expression of CD133 and TIE2 at the aortic endothelium of the human embryo, it is impossible to identify the function of this co-expression. It can be speculated that this co-expression supports the hemogenic role of the embryonic aorta endothelium, which gives endothelial cells the ability to differentiate both in endothelial precursor and hematopoietic precursors, a theory already accepted at the level of the aortic endothelium in the aorto-gonado-mezodermic area but not described so far at the level of the thoracic aorta.
